# Poly-Alanine-ε-Caprolacton-Methacrylate as Scaffold Material with Tuneable Biomechanical Properties for Osteochondral Implants

**DOI:** 10.3390/ijms23063115

**Published:** 2022-03-14

**Authors:** Nicole Hauptmann, Johanna Ludolph, Holger Rothe, Jürgen Rost, Alexander Krupp, Jörg Lechner, Svenja Kohlhaas, Manuela Winkler, Benedikt Stender, Gerhard Hildebrand, Klaus Liefeith

**Affiliations:** 1 Institute for Bioprocessing and Analytical Measurement Techniques e.V. (iba) Rosenhof, 37308 Heilbad Heiligenstadt, Germany; nicole.hauptmann@iba-heiligenstadt.de (N.H.); johanna.ludolph@iba-heiligenstadt.de (J.L.); holger.rothe@iba-heiligenstadt.de (H.R.); juergen.rost@iba-heiligenstadt.de (J.R.); gerhard.hildebrand@iba-heiligenstadt.de (G.H.); 2 Multiphoton Optics GmbH, Friedrich-Bergius-Ring 15, 97076 Wuerzburg, Germany; alexander.krupp@multiphoton.de (A.K.); joerg.lechner@multiphoton.de (J.L.); svenja.kohlhaas@multiphoton.de (S.K.); manuela.winkler@multiphoton.de (M.W.); benedikt.stender@multiphoton.de (B.S.)

**Keywords:** osteochondral implant, scaffold, biodegradable polymer, biomechanics, Two-Photon-Polymerization, Poly-Alanine-ε-Caprolacton-Methacrylate, amino acid

## Abstract

An aging population and injury-related damage of the bone substance lead to an increasing need of innovative materials for the regeneration of osteochondral defects. Biodegradable polymers form the basis for suitable artificial implants intended for bone replacement or bone augmentation. The great advantage of these structures is the site-specific implant design, which leads to a considerable improvement in patient outcomes and significantly reduced post-operative regeneration times. Thus, biomechanical and biochemical parameters as well as the rate of degradation can be set by the selection of the polymer system and the processing technology. Within this study, we developed a polymer platform based on the amino acid Alanine and ε-Caprolacton for use as raw material for osteochondral implants. The biomechanical and degradation properties of these Poly-(Alanine-co-ε-Caprolacton)-Methacrylate (ACM) copolymers can be adjusted by changing the ratio of the monomers. Fabrication of artificial structures for musculo-skeletal tissue engineering was done by Two-Photon-Polymerization (2PP), which represents an innovative technique for generating defined scaffolds with tailor-made mechanical and structural properties. Here we show the synthesis, physicochemical characterization, as well as first results for structuring ACM using 2PP technology. The data demonstrate the high potential of ACM copolymers as precursors for the fabrication of biomimetic implants for bone-cartilage reconstruction.

## 1. Introduction

In the last decades, numerous efforts have been made for the treatment of osteochondral defects and bone diseases by means of musculo-skeletal tissue engineering [1]. Articular cartilage defects can exhibit many different sizes and shapes and vary in depths. In the most severe cases, the defects include the underlying subchondral bone. Critical size defects need invasive surgical interventions to reconstruct the structural integrity of the damaged tissues and protect the joint from developing osteoarthritis. Among all innovations, autologous implants serve as the “golden standard” in clinical practice, due to the lack of immunological or foreign body reactions caused by the implants. Nevertheless, autologous implantation shows many disadvantages, such as the necessity of two surgical interventions, limited availability of suitable donor tissue and a significant morbidity [2]. A second surgical option is the use of allografts or xenografts. However, it should be noted that these materials often cause immunological problems due to serious foreign body reactions in the host bone, as well as a significantly reduced regeneration capacity [3]. Thus, the establishment of new materials and technologies for bone and cartilage regeneration is of great interest for reducing pain in patients as well as for social-economic reasons. Nowadays, the costs for the therapy of a patient with critical size defect lie between €10,000 and €100,000, and there are about 1 million bone grafting surgeries each year in Europe and about 2.2 million surgical interventions worldwide. It is of value to note that, in most cases, autologous implants are used [1]. Therefore, the development of new therapeutic approaches represents an important aspect to take into account.

In the last decades, regenerative medicine and tissue engineering have gained enormous interest for the development of implants for bone and cartilage repair. Thus, many research groups focus on the development of new technologies for scaffold design and production. In Tissue Engineering, the scaffolds should have appropriate biomechanical and biochemical properties and an ECM-analogous macroporous architecture enabling the transport of nutrients, metabolites and cytokines to ensure both the cell–cell and the cell–ECM communication inside the scaffold. The scaffold should have a tissue-adapted degradation rate and osteoinductive/osteoconductive properties. Ideally, the scaffold should support the defects mechanically until new bone is formed. Natural bone and cartilage are characterized by an anisotropic structure; thus, the scaffold should consist of a hierarchically organised architecture [4,5]. Another important point is that growth factors should be incorporated inside the structure to enhance osteogenesis, chondrogenesis and angiogenesis [3,5,6]. In many cases, rapid prototyping methods, like stereolithography, fused deposition modelling, selective laser sintering or electrospinning are used for the establishment of artificial bone or cartilage scaffolds [7]. These technologies fail in generating a hierarchical structure with well-defined pore sizes and pore distribution because of their limited spatial resolution. 

Among all rapid prototyping methods, Two-Photon Polymerization (2PP) plays an outstanding role because of the excellent spatial resolution down to the nm range and its inherent 3D capability, enabled by the underlying non-linear excitation process. Triggered by Two-Photon Absorption (2PA), the polymerization occurs only in the confined focal volume (Voxel). The size of the induced voxel is dependent on laser power, exposure time, optical set-up and the precursor material in connection with a suitable photoinitiator [8,9]. In this way, any 3D structure can be created by scanning the focal volume through a photosensitive material. Because of the high resolution and the flexibility of 2PP technology, it is in fact possible to modulate the scaffold properties to match those of the native osteochondral tissue [10]. 

As materials for 2PP, usually methacrylated or acrylated poly-/oligomers in combination with an appropriate photoinitiator with a high two-photon absorption rate were used. Bearing in mind the large number, specificity and complexity of suitable photosensitive materials, it is intuitive that these materials should be biocompatible and biodegradable with a controlled degradation rate. The degradation products should not (i) induce a toxic or immunogenic response, or (ii) disturb the physiological healing process due to potential foreign body reactions. Usually, photosensitive polymers are divided into synthetic polymers, such as methacrylated or acrylated Polylactide [11], Polycaprolacton [12], Polyethyleneglycole [13], Polyglycolide and their copolymers [10,14], and natural polymers, such as collagen [15], gelatine [16], hyaluronic acid [17] or chitosan [18]. The advantages of synthetic polymers are their defined mechanical and chemical properties, while natural polymers are advantageous due to their inherent bioactivity. Thus, the ideal way is to combine both synthetic and natural polymers to gain a tissue-analogue mechanical support of the structure with adjustable bioactivity and degradation kinetic. It is generally accepted that polylactide-containing polymer systems form lactide acid during degradation, which often results in a decrease of the local pH value, leading to acetolysis [19]. 

Within this study we developed a polymer platform based on the amino acid alanine and ε-caprolacton to replicate the complex 3D architecture of the osteochondral ECM for bone and cartilage reconstruction. The use of caprolacton in biomedical applications is FDA (Food and Drug Administration) approved. By changing the ratio of alanine and caprolacton, the stiffness and degradation rate of the final copolymer can be adjusted. The methacrylated Poly-Alanine-ε-caprolacton was processed with 2PP to fabricate any 3D object without any structural restrictions. By changing the parameters for 2PP, the porosity and the stiffness of the scaffold can be tailored to mimic the natural ECM architecture with high fidelity. 

## 2. Results and Discussion

### 2.1. Synthesis of 2PP Processable Polymers

Keeping in mind the fact that, so far, no method has been able to consistently achieve a complete regeneration of osteochondral defects with respect to the native tissue, new approaches based on tissue engineering were introduced. Commonly used surgical techniques for treatment are, for example, matrix-induced autologous chondrocyte implantation (MACI) or matrix-induced autologous stem cell implantation (MASI). As part of these strategies, the use of cell-seeded or non-cell-seeded three-dimensional scaffolds provides support for tissue growth and guides new tissue formation analogue to the native ECM. It seems to be clear that, besides bioengineering and cell biology, material science plays a pivotal role. Common materials used for loadbearing implants (e.g., metals/oxid ceramics) exhibit several disadvantages, where the most important one is the stiffness mismatch between the implant material and the natural osteochondral tissue, leading to limited implant lifetime and implant loosening. Therefore, current research focuses on the establishment of new biodegradable materials where the host tissue rebuilds the implant material by enzymatic and hydrolytic degradation and produces its own ECM as part of its metabolic activity. Promising materials for regeneration of osteochondral defects seem to be poly(ester amides), whose chemical structure consists of ester and amide groups leading to a precisely adjusted balance between degradation kinetics and mechanical stiffness. The excellent mechanical properties of these polymers are based on the strong hydrogen bonding between the amide groups in the polymer, whereas an increase of ester blocks in the polymer weakens the stiffness [20]. Within this study, we developed a polymer platform, based on amino acid alanine and ε-caprolacton, for the preparation of osteochondral implants by Two-Photon Polymerization (2PP). Within the Poly-(Alanine-co-ε-caprolacton) polymers, the content of the monomers was varied to generate a material platform with defined mechanical and biodegradation properties. The polymer degrades in non-toxic monomers, such as caprolacton and alanine. Methacrylation of the endgroups of the polymer leads to highly crosslinkable photoresists with excellent suitability for 2PP.

**Figure 1:**
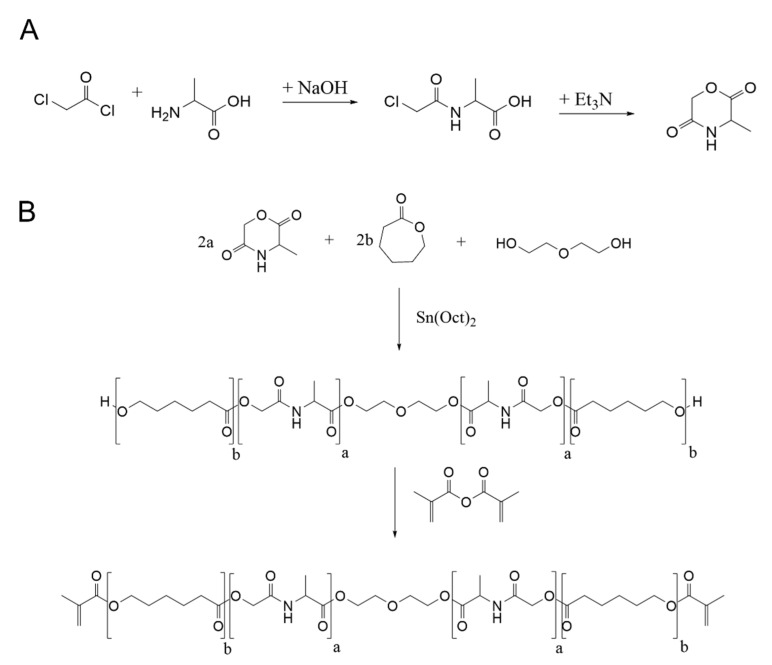
Synthesis of (**A**) Cyclic monomer: N-Morpholino-2,5-dion and (**B**) Poly-(Alanine-ε-Caprolacton)dime thacrylate (a…2, 4, 6, 8 and b…8, 6, 4, 2).

In Figure 1, the reaction scheme for the synthesis is shown. First of all, a cyclic alanine derivate was synthesized for later use in ring opening polymerization. This occurred by a two-step reaction of N-Chloroacetylchlorid with alanine followed by cyclization to N-Morpholine-2,5-dion. This synthetic route is commonly used to generate various cyclic peptide derivatives for further polymerization reactions [20,21,22]. Both steps were characterized by ^1^H-NMR spectroscopy. Modification of alanine was detected by the newly formed singlet for CH_2_ group at 4.05 ppm, and successful cyclization of N-Chloroacetylalanine to cyclic monomer was shown by formation of two new doublets between 5.3–5.4 ppm and a shift of the peak for the CH_3_ group to 1.6 ppm. Then, cyclic monomer was polymerized to photocrosslinkable Poly-(Alanine-ε-Caprolacton)- dimethacrylate by cationic ring opening polymerization using diethylene glycol as bifunctional crosslinker and Sn(II)EtHex as anorganic catalyst. Four copolymers were synthesized which differed in the ratio of cyclic alanine derivate (20, 40, 60, 80 mol%) and caprolacton (80, 60, 40, 20 mol%) to establish a polymer platform with tailor-made mechanical and biodegradation properties. For the synthesis of the different copolymers, the reaction procedure had to be modified because of the melting and boiling points of the monomers. Thus, copolymers with higher alanine (60%, 80%) content were synthesized at a higher temperature (150 °C), whereas the reaction time was reduced from 64 h to 20 h. The resulting polymers were methacrylated to obtain highly reactive precursors for 2PP.

**Figure 2:**
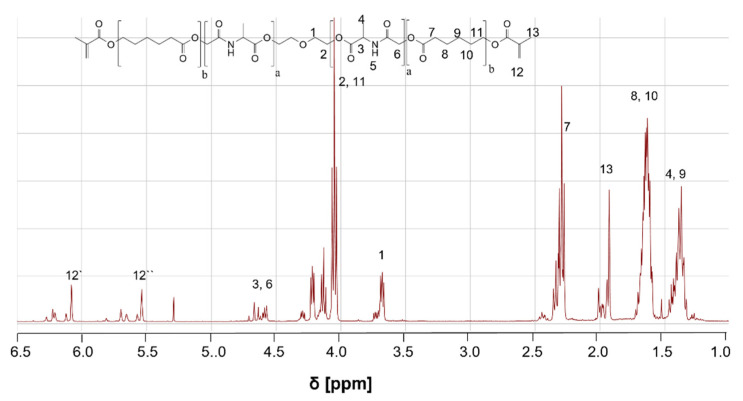
^1^H-NMR Spectra of Poly-(Alanine-ε-Caprolactone)dimethacrylate 2:8.

All ACM copolymers were characterized by ^1^H-NMR spectroscopy. In Figure 2, the ^1^H-NMR spectra of ACM 2:8 is shown. The broad peak for the diethyleneglycol crosslinker at 3.7 ppm, the signal for the alanine block (4.6–4.7 ppm) and the peaks for the caprolacton block in the copolymer (1.5–1.7 ppm; 2.3–2.4 ppm) are clearly visible. Furthermore, one can see the successful methacrylation of the polymer by the newly formed peaks for the methylene group at 5.5 ppm and 6.1 ppm. Splitting of the signal for the methylene group is due to the interaction of one proton with the neighbouring ester group, leading to a stabilized mesomeric structure. The diethylenglycol peak is used as reference peak for further calculation of degree of polymerization, real ratio of alanine and caprolacton content in the copolymer and the degree of methacrylation. Furthermore, the molar mass was determined using the NMR spectra. 

**Figure 3:**
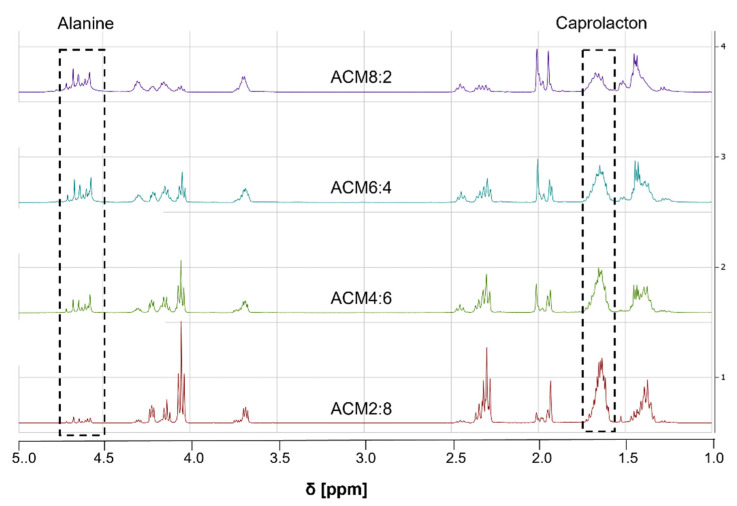
1H-NMR Spectra of Poly-(Alanine-ε-Caprolactone)dimethacrylate with different ratios of alanine and caprolacton.

Figure 3 and Table 1 summarize the ^1^H-NMR spectra of ACM 8:2, ACM 6:4, ACM 4:6 and ACM 2:8 regarding the integral values of the alanine and caprolacton peak. Thus, when increasing alanine content in the reaction from 20 mol% to 80 mol% the integral of the peak for alanine increased from 1.3 to 5.4 (Table 1). Correspondingly, a decrease of the area of the caprolacton peak (7.2–2.0) was observed. The conversion of the alanine monomer, which is defined as the ratio of the real alanine content measured by the integral of the alanine peak and the theoretic value, is between 65% and 72%. In contrast to that, the conversion of the caprolacton monomer is significantly improved and lies above 90%. Nevertheless, the conversion ratio for cyclic alanine monomer was excellent when compared with data recently published. John et al. [23] showed that the yield of the polymerization of cyclic serine monomer with lactide decreased from 85% to 60% with increased cyclic serine monomer from 2.5 mol% to 20 mol%. Polymerization with caprolacton also showed poor conversion of 63% and 52%, regarding a serine monomer content of 5 mol% and 10 mol%. In another study by Ouchi et al. [24], copolymers of cyclic glycin-lysin derivate (5–75 mol%) with lactide were synthesized and conversion ratios of the amino acid derivate between 86% and 57% were observed. Obviously, the conversion is dependent on the amount of cyclic peptide. Thus, an increase of cyclic peptide in the reaction results in a lower conversion rate. The conversion of cyclic alanine monomer fits the literature data, and optimization of the synthetic route for ACM copolymers with higher amide content results in an increase in conversion of cyclic monomer. The results show unambiguously that we could successfully synthesize ACM copolymers with different ratios of the amide and ester block leading to defined, tailor-made stiffness and degradation properties. 

### 2.2. Physicochemical Properties of the Polymers

One of the most important aspects for 2PP structuring is a specific viscosity of the precursor to avoid undesired motion of structures during fabrication. In 2PP, the sample is positioned on a precision stage which moves through the focus of the laser beam. Highly viscous resins are advantageous for 2PP to reduce fluid dynamics which are caused by the acceleration of the axes [25]. The viscosity of the resin correlates with the kinetics of the polymerization process by a reduction of oxygen diffusion in highly viscous resins. This leads to a decrease of radical quenching [26]. Caused by the limited radical diffusion in highly viscous resins, the voxels and line width are smaller than in resins with lower viscosity [25]. There are two parameters which are important for polymerization: the polymerization threshold and the damage threshold. The first one is dependent on the characteristics of the photoinitiator and the concentration of the precursor, while the second one is dependent on the intrinsic properties of the precursor. The processing window between the two thresholds is called dynamic range. Studies show that the dynamic range increases for more viscous resins [25]. Additionally, more viscous resins simplify sample preparation, because often only small sample volumes are needed for 2PP, which can be placed very easily right below the lens. Thus, 2PP resins are usually highly viscous resins or semi-solid formulations [27,28]. 

**Figure 4:**
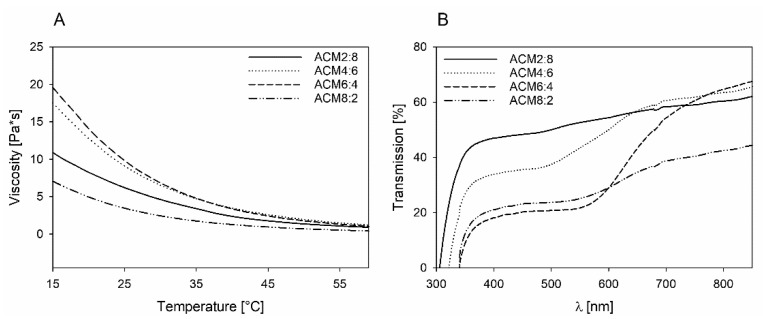
Physicochemical properties of ACM. (**A**) Temperature-dependent viscosity and (**B**) UV-Vis Spectra.

Because of these reasons, the viscosity of the polymer was analyzed in dependence of the temperature using a MCR rheometer. The results are shown in Figure 4A. The ACM derivatives are highly viscous solids at room temperature, and viscosity decreases with increasing temperature. The results also show that there is a clear dependency of viscosity on the caprolacton content. Thus, a reduction of caprolacton content leads to a higher viscosity, which could be due to intermolecular hydrogen bonding between the polyamide chains. The ACM derivative with the highest alanine content showed a contrary behavior of the temperature-dependent viscosity. Thus, for the viscosity of ACM 8:2, the lowest values in comparison with the other polymers were observed. For 2PP it was demonstrated that the energy threshold decreases with increased viscosity. In a study by Zandrini et al. it was shown that the power needed for initiating polymerization is up to 4.5 fold higher for precursors having a lower viscosity. It has been found that the best polymerization conditions were obtained for a viscosity of 10.4 Pa*s, whereas the dynamic range increased exponentially until it reached a plateau at viscosities higher than 0.1 Pa*s [25]. Thus, there should be a broad dynamic range for 2PP for all polymers. 

For establishing photoresists for 2PP, optical properties like the transparency of the material at the wavelength of the laser used is important for polymerization in the focal volume. An excellent light penetration during the polymerization process results in an optimal 2PP structuring and a high quality of the fabricated structures [29]. It is therefore common to measure UV-Vis spectra of the ACM copolymers (Figure 4B). Results show that the light penetration increased with increasing wavelength from 300 nm to 880 nm. At 400 nm, transmission of the polymers was between 18% and 47% depending on the content of cyclic alanine, whereas a higher amount of amino acid led to a reduction in light penetration. At a wavelength of 800 nm, transmission increased to 42% and 64%, and the dependence of the light penetration regarding the alanine content was negligibly small, with the exception of ACM 8:2. For the copolymer with the highest amide content, the IR penetration depth was significantly reduced compared with the other ACM copolymers. 

**Figure 5:**
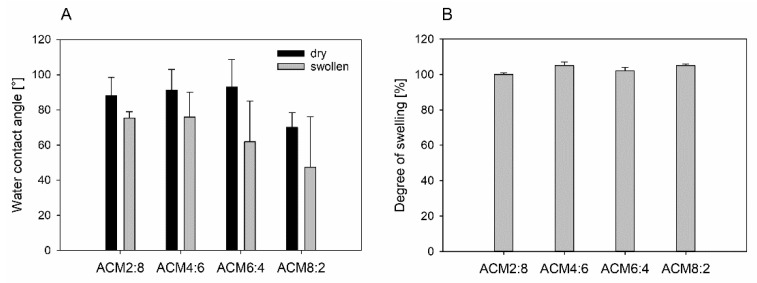
(**A**) Water contact angles of the different ACM copolymers in dry and hydrated states (data are presented as mean ± standard deviation for n = 90) and (**B**) Swelling behavior of the ACM copolymers.

It is generally accepted that material properties, like wettability, influence the initial blood-implant interactions, which are mainly the adsorption of proteins on the implant surface. The adsorption of proteins is controlled by an extremely complex superposition of protein/surface, protein/protein, protein/water, and water/surface interactions [30]. Thus, an increased surface energy and wettability leads to a decrease of surface-induced protein adsorption and protein denaturation and a decrease of cell adhesion, and affects cell growth [31,32]. It was already shown in literature that a dense fibrin network is preferably formed on hydrophilic surfaces, leading to tissue integration, promotion of osseointegration and reduction of healing processes [33]. Therefore, hydrophilicity is a critical property when designing materials for scaffolds because it determines the interaction of cells and dissolved proteins with the artificial extracellular matrix [34]. Wettability of the ACM copolymers was determined for the dry samples and the swollen material (Figure 5A). To simulate cell culture conditions, the ACM discs were incubated for 72 h in PBS buffer at 37 °C. Results of the contact angle measurement show that the wettability increases for the swollen samples (47–76°) in comparison to the dry ones (70–93°). Furthermore, there is an influence of the amount of alanine on the contact angle. Thus, an increase of amide groups inside the polymer material leads to an increase of wettability. This could be due to the nature of the amide groups, which form intramolecular hydrogen bonds between the polymer chains, leading to a more hydrophilic character of the material. This effect is not observed for the dry samples because of reduced hydrogen bonding ability in the dry state.

In a study by Samadian et al., hybrid scaffolds consisting of polycaprolacton, polylactide and gelatine nanofibers with water contact angles between 81° and 109° were prepared. The results show higher cell proliferation on scaffolds with a lower contact angle [34]. Furthermore, hydrophilicity of polycaprolacton/collagen and polycaprolacton/gelatine scaffolds have shown a large effect on cellular adhesion, proliferation and viability [35]. Thus, when comparing our results with the literature data, contact angles fit the region to promote adhesion and proliferation of cells from the osteoblastic lineage. Another important material characteristic is the swelling behavior. This has an influence on form and structure stability of the final scaffold. For polymers which absorb huge amounts of water, the structure which is processed by 2PP must be adjusted according to the degree of swelling or shrinkage. Swelling behavior after incubating the ACM discs in PBS for 72 h is shown in Figure 5. Degree of swelling is quite low for all samples and lies between 99% and 102%. By definition, it must be noted that a degree of swelling of 100% corresponds to absolutely no deformation as a consequence of water absorption. Thus, it can be concluded that the ACM copolymers show no swelling in aqueous solution.

### 2.3. Viscoelastic Properties of ACM Copolymers

For the establishment of a new polymer platform for osteochondral reconstruction, the viscoelastic properties of the scaffold and the scaffold material should be adjusted to the natural osteo- and chondrophase. Thus, the material should mimic the natural environment of the implant, which means that the implant material should have an appropriate stiffness to enhance proliferation and differentiation of mesenchymal stem cells. This means that the closer the mechanical properties of the scaffold to those of the replaced natural tissue, the higher is the expected healing efficiency. Therefore, it seems inescapable that materials which are not adapted to the properties of the tissue around the implant can inhibit proper postoperative integration and regeneration of the tissue [36]. 

**Figure 6:**
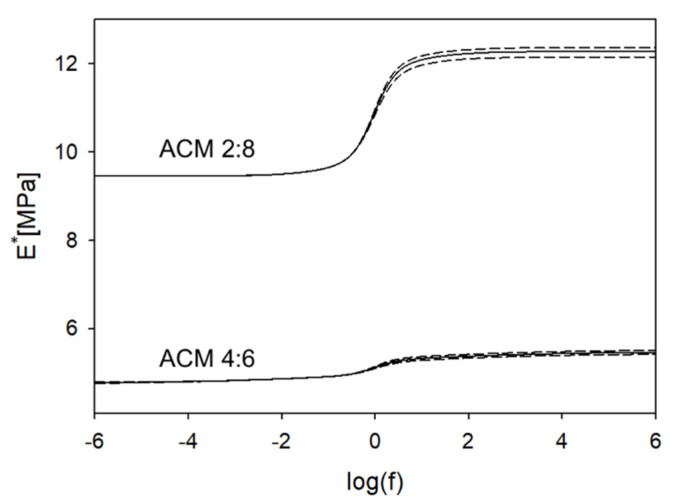
Master curves of the complex viscoelastic modulus for ACM 2:8 and ACM 4:6; solid lines: median value, dotted lines: 50% quantile.

Consequently, a comprehensive analysis of the mechanical behavior of the different copolymers strictly based on the theory of viscoelasticity was performed. In dynamic mechanical spectroscopy, the time–temperature superposition principle is often used to model the behavior of materials at a defined range of frequencies to generate a so-called master curve. Due to the limited thermostability of the polymer system used in this study, we calculated the viscoelastic properties based on an evolutionary fitting strategy of stress relaxation measurements as described recently [37]. The resulting master curves (complex modulus vs. frequency) of the two materials tested, obtained from stress relaxation measurements, are shown in Figure 6.

**Figure 7:**
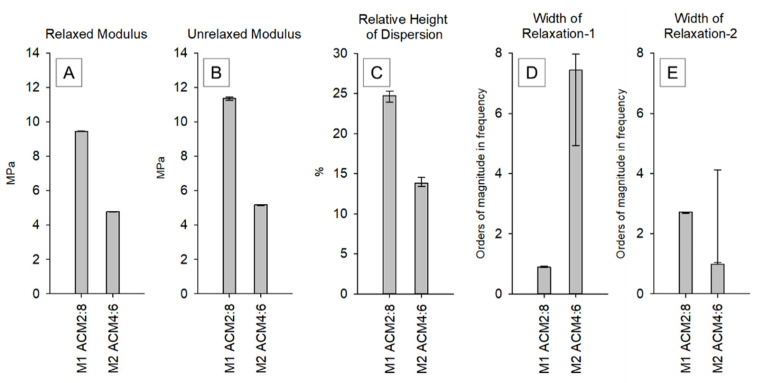
Characteristic values for both copolymers; (**A**) relaxed modulus at f = 0; (**B**) unrelaxed modulus at f = ∞; (**C**) height of global dispersion relative to unrelaxed modulus; (**D**) width of relaxation region-1; (**E**) width of relaxation region-2.

It is obvious that an increased caprolacton content leads to a generally stiffer material (Figure 7A,B). However, it is very remarkable that an increased proportion of alanine in the copolymer shows a lower dispersion step and, following that, a less-intensive relaxation behavior which renders the copolymer more elastic (Figure 7C). The position of the dispersion centers for both copolymers is in the physiologically relevant range around 1Hz. Interestingly, the higher content of alanine led to a widening of the first relaxation region by several orders of magnitude (Figure 7D).

Polycaprolacton is a partially crystalline polymer up to approximately 60 °C, which explains why the caprolacton-rich copolymers are generally stiffer and show a complex modulus of approx. 10–12 MPa. The polar character of the alanine leads to the formation of hydrogen bonds within the polymer, which can be an additional explanation for the observed shift of the mechanical behavior of the caprolacton-rich polymer and the alanine-rich polymer.

In our previous work based on finite element analysis, we were able to show that the stiffness of the porous scaffolds is approximately 1/20 of the stiffness of the cylindric monolithic samples [10]. Thus, the calculated stiffness for the 2PP produced osteochondral implants can be expected to be in the range of 400 kPa for ACM 2:8 and 230 kPa for ACM 4:6. The stiffness of the other ACM derivatives could not be measured because of the strongly reduced UV light penetration when increasing the amount of alanine, which makes a light-induced polymerization of the cylindric monolithic samples by UV nearly impossible. Corresponding transmission experiments showed that light penetration at 400 nm was 22% for ACM 6:4 and 20% for ACM 8:2.

Furthermore, it is of limited possibility to compare published mechanical properties with each other, since the measurement methodology is often very different. Another critical point that complicates the comparability of results is the fact that most polymeric biomaterials exhibit a pronounced frequency-dependent viscoelastic behavior which is less considered in respective standards, for example ASTM 1621–10:2010 or ISO 844. Our strategy to solve these problems focuses on characterizing the viscoelastic behavior by means of relaxation measurements. The development and evaluation of this methodology we recently published elsewhere [37]. 

Nevertheless, if one tries to compare different polymeric materials and scaffold architectures, the material platform we present is within the wide range of published mechanical properties for scaffold materials in bone healing applications. Table 2 compares some published material and scaffold platforms with the respective measurement methodology discussed in the field of bone healing applications.

Thus, Kampleitner et al. showed that Poly-(Lactide-co-ε-Caprolacton)-Scaffolds with a stiffness between 100–300 kPa showed excellent bone healing properties [38]. In another study by Häussling et al., poly-(2-hydroxyethyl methacrylat) cryogels with different kinds of proteins were used to increase the bone healing effect. The cryogels with a stiffness between 60–200 kPa led to an increase in the differentiation of mesenchymal stem cells [39]. In contrary to that, Hong et al. established hybrid polymers with cellulose and Poly-ε-Caprolacton and showed excellent biocompatibility when cultivating MC3T3-E1 preosteoblasts on the scaffolds [40]. Portan et al. showed that polylactide scaffolds with a tensile strength of 10–18 MPa induce differentiation of osteoblasts [41]. 

### 2.4. PP Structuring of ACM 

In the following, preliminary structuring results are presented demonstrating the general capability to process the ACM polymer system via 2PP. As material, ACM 2:8 supplemented with 2 wt.% of the photoinitiator Irgacure^®^369 was used. Details concerning sample preparation, setup and equipment used for analysis are discussed in chapter 3.8.

**Figure 8:**
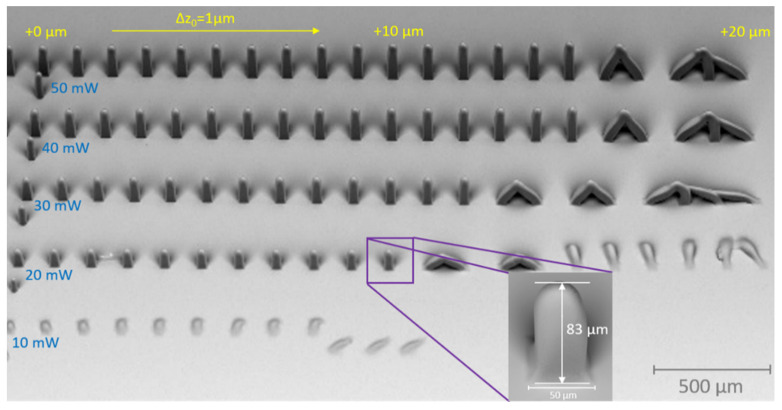
SEM (scanning electron microscopy) images of cuboids fabricated by 2PP with variation of fabrication parameters for ACM 2:8. For each row the laser power was modified (blue labeling). The focusing offset with respect to the detected interface is varied within each row (yellow labeling). In the inset, a close-up of one of the cuboids is shown. Scale bars are 500 µm for overview image and 50 µm for close-up.

Figure 8 displays typical results of a parameter search process for 2PP structuring. For a structure to remain mechanically stable after the development process, the polymerized material should form a stable contact with the substrate. This requires that the focal point of the laser beam must be partially submerged in the substrate. Hence, the ascending scan technique was applied at varying process parameters with the aim of finding the optimal interface z_0_-position between glass substrate and ACM, as well as to study the shrinkage behavior of ACM. Small cuboids with target dimensions of 50 × 50 × 30 µm^3^ were fabricated in a layer-by-layer approach, with a slicing and hatching distance of 1 µm each, and a scanning speed of 150 mm/s (Figure 8). Within each row from left to right, the writing offset position z_0_ of the structures with respect to the detected interface was increased from −1 µm to +20 µm in increments of 1 µm. In the vertical direction, the laser power was increased in steps of 10 mW, starting at 10 mW at the bottom.

The height of the structures increases with increasing laser power as a result of the longer axial voxel. From left to right, the z_0_-offset of the cuboids is increased in steps of 1µm and results in collapsed structures at too-large z_0_-offset positions (+11 µm to +16 µm) as their adhesion to the interface decreases. The optimal z_0_-offset position with respect to the detected interface is in the range of +10 µm to +15 µm. At a laser power of 10 mW, no stable structure could be achieved.

As expected, the height of the structures does not match with the target value of 30 µm. As measured via laser scanning microscopy, the height of stable structures increases from about 83 µm, for cuboids fabricated with a laser power of 20 mW (shown in the inset of Figure 8), to about 175 µm for an applied laser power of 50 mW. The main reason for this discrepancy between target height and actual height is the low numerical aperture of NA = 0.07 of the f-theta objective used, inducing an enlarged voxel in the axial direction. The inherent doubled Rayleigh length, calculated to be about 75 µm in the z direction, induces a very large axial focal dimension, thereby triggering enlarged axial voxel dimension of larger than 75 µm, dependent on applied laser power and the investigated copolymer. Considering the largest cuboid of h_max_ = 175 µm (P = 50 mW), the voxel’s size in the axial direction can be estimated at 2 × (h_max_ − h_target_) = 290 µm with h_target_ = +30 µm. The z_0_-offset was not considered in this simple approximation since the structure has to be connected to the substrate.

However, since the primary goal is the fabrication of large scaffolds with dimensions of 10 mm in height and 7 mm in diameter, including macropores of about 400–1100 µm and micropores of about 5–20 µm in the xy direction, the pronounced axial voxel dimension is still acceptable. On the contrary, this elongated voxel in the zdirection is even beneficial for this application since it reduces the fabrication time tremendously, as larger slicing distances can be chosen while still achieving a stable structuring result [42]. This is a major benefit compared to conventional 2PP approaches and enables a higher throughput.

**Figure 9:**
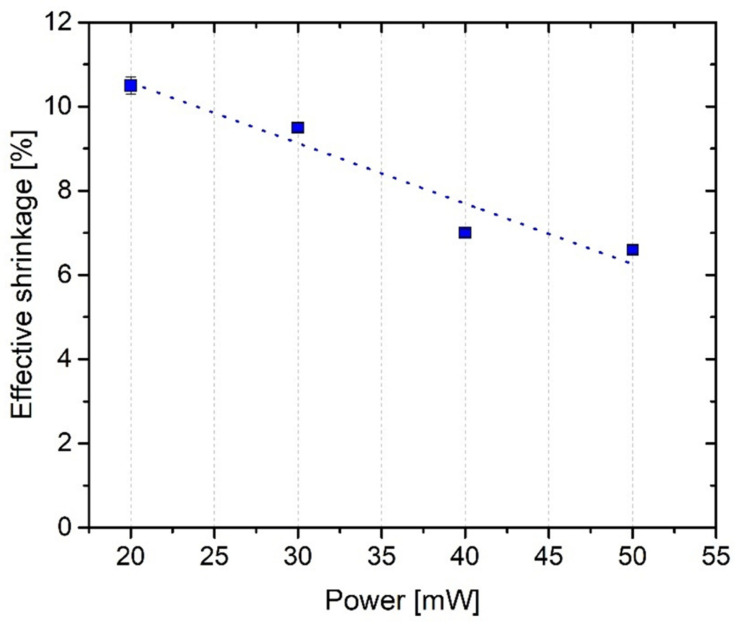
Effective shrinkage of cuboids in ACM 2:8 dependent on the applied laser power reveals the occurrence of a non-constant shrinkage behavior. The dotted line is a guide-to-the-eye.

To analyze the shrinkage behavior of ACM 2:8, the resulting lengths and widths of the cuboids were determined from SEM (scanning electron microscopy) images and compared to the default value of 50 µm. For each laser power resulting in stable structures, the lengths and widths of 10 structures were averaged. Figure 9 displays the results, thereby exhibiting a linear dependence of the effective shrinkage on the laser power from about 10.5% to about 6.6% with increasing laser power. To explain the reasons for this power-dependent behavior, two counteracting effects have to be considered. The material-based shrinkage and its counteracting effect by a laser-power-dependent voxel size lead to an effective shrinkage. Within the measurement, these two effects cannot be separated. With increased voxel size the outer dimensions of the cuboids continuously increase during fabrication, thereby resulting in a decrease of the effective shrinkage. To compensate for the shrinkage, an adaptation of the design is necessary. To separate the power-dependent enlargement of the voxel from pure material shrinkage, further experiments are necessary which are not within the scope of this work.

Based on these impressive first results, it is certainly not surprising that the fabrication of larger structures in the mm range will be the focus of the next steps. As already mentioned, the fabrication of biphasic osteochondral scaffolds which were already successfully fabricated using a Poly-((D,L)-Lactide-co-ε-caprolacton)-dimethacrylate material system [43] shall be transferred to the system Poly-(Alanine-co-ε-Caprolacton)-dimethacrylate, thereby avoiding the appearance of acid degradation products.

### 2.5. Proliferation of Mesenchymal Stem Cells

**Figure 10:**
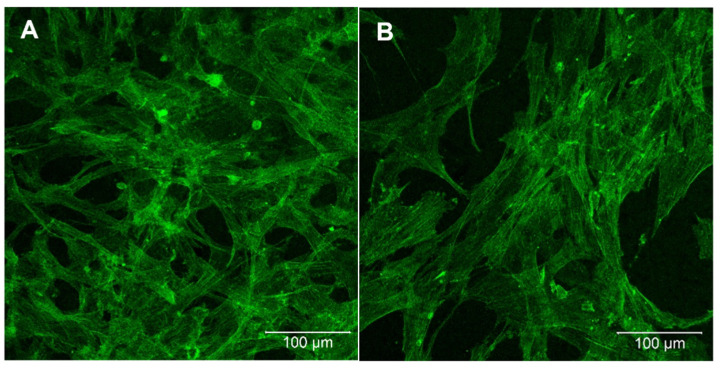
Confocal fluorescence images of bone marrow mesenchymal stem cells (BMSCs) cultured on ACM (**A**,**B**). (**B**) was coated with Poly-L-Lysin/Heparin to avoid any hemostatic reaction after blood contact. Green: Phalloidin488. Scale bars are 100 µm each.

A major aspect and one of the most important design criteria of photosensitive polymers suitable for 2PP is their intrinsic biocompatibility. As part of a first approach, we analyzed whether mesenchymal stem cells (MSCs) adhere on the ACM material. Therefore, polymer discs were fabricated as described in Section 3.6. To evaluate whether the non-modified synthetic polymer itself can enhance adhesion and proliferation of the cells, or whether a biological stimulus was needed, half of the samples were modified with a heparin coating using the well-known layer-by-layer (LbL) method. After an incubation time of 72 h, cells were stained with Phalloidin488 and the morphology of the cells was analyzed using confocal fluorescence microscopy (Figure 10). The results show an excellent cell adhesion on the ACM samples, with high cell spreading. Comparing the uncoated and heparin-coated samples, one can see that the total number of cells on the uncoated samples is much higher than on the samples with heparin coating, indicating the high biocompatibility of the ACM polymer. Keeping in mind that the heparin layer (i) possesses a high affinity to water, which can inhibit an initial cell adhesion, and is, (ii) regardless of its water affinity, necessary to avoid any hemostatic reaction after blood contact, the data for both material modifications are within the expected range. Independent of the heparin coating, cells form a nearly confluent cell layer already after 3 d cultivation. 

## 3. Materials and Methods

### 3.1. Materials

L-Alanine, Chloroacetylchloride, Sodium Hydroxide, Hydrochloric acid Dimethylformamide, Diethylether, Triethylamine, Ethylacetate, Magnesiumsulfate, Chloroform, Isopropanol, Diethylene glycol, Caprolacton, Stannus(II)-Ethylhexonate, Methacrylic acid anhydride, Methacrylic chloride, Dichlormethane, heptane, hexane, acetone, sodium chloride, phosphate buffered saline were purchased from Sigma Aldrich, Taufkirchen, Germany.

### 3.2. Synthesis of 3-Methyl-morpholine-2,5-dione

For the synthesis of the N-Chloroacetylalanine, L-Alanine (35.64 g, 0.4 mol, 1 eq) was dissolved in 75 mL deionized water and 100 mL 4M sodium hydroxide at −5 °C in a three neck round bottom flask. To this solution, 75 mL Diethylether was added. Then, a mixture of 35 mL (0.44 mol, 1.1 eq) Chloroacetylchloride in 50 mL Diethylether was prepared and 150 mL 4M NaOH was filled in a second dropping funnel. Both solutions were added simultaneously drop-wise to the alanine solution over a time period of 30 min, while keeping the pH value constant at 11 as well as the temperature at −5 °C. The resulting mixture was stirred 2 h at 0 °C and then allowed to warm up to room temperature. The ether phase was removed and the water phase was adjusted to pH 1 using 4M HCl. After that, the water phase was extracted four times against 200 mL Ethylacetate and the combined organic fractures were washed with 200 mL saturated NaCl, followed by drying the organic phase with MgSO_4_. Then, the solvent of the filtered solution was evaporated using a rotary evaporator and the product was recrystallized two times with Ethylacetate. The product was dried overnight under vacuum at 50 °C (Yield: 70%).

^1^H-NMR (D_2_O): δ [ppm]: 1.33–1.34 (d, 3H, CH_3_), 4.05 (s, 2H, CH_2_), 4.28–4.30 (q, 1H, CH) 

For the cyclization step, 12 g (0.073 mol, 1 eq) of N-Chloroacetylalanine was added to a 500 mL two neck round bottom flask and dried under vacuum for 1 h. The educt was dissolved in 300 mL dimethylformamid under Argon atmosphere. Subsequently, the flask was evacuated and filled with argon three times. Then, triethylamine was added to the solution and the mixture was stirred at 100 °C for 8 h. After that, the oil bath was removed and the reaction mixture was cooled down to room temperature. The Et_3_N*HCl salt was filtered off and the solvent was evaporated at 5 mbar and 70 °C. The resulting product was recrystallized two times with isopropanol. (Yield: 50%)

^1^H-NMR (D_2_O): δ [ppm]: 1.39–1.41 (d, 3H, CH_3_), 4.30–4.34 (q, 1H, CH), 4.71–4.86 (2d, 2H, CH_2_) 

### 3.3. Synthesis of Poly-(alanine-co-ε-caprolacton)-dimethacrylate (ACM 2:8 and ACM 4:6)

Polymerization of the cyclic peptide for ACM 2:8 was performed as follows: 3-Methyl-morpholine-2,5-dion (2.43 g, 18.8 mmol, 2 eq), ε-Caprolacton (8.35 mL, 75.4 mmol, 8 eq) and Diethyleneglycol (0.89 mL, 9.4 mmol, 1 eq) were added in a flame-dried two-neck round-bottom flask. The educts were dried under vacuum for 2 h and the flask was flushed with argon. Then, the flask was heated to 120 °C and Sn(II)EtHex (0.3 mL, 0.9 mmol, 0.1 eq) was added under argon atmosphere using a sealed flask under vacuum conditions. The reaction mixture was stirred at 120 °C for 64 h. After that, the flask was cooled down to 60 °C and the product was dissolved in 14 mL methacrylic anhydride (94.2 mmol, 10 eq). Methacrylation of the polymer was performed for 20 h at 60 °C under vigorous stirring. Then, the product was precipitated five times in cold hexane and dissolved in 200 mL dichlormethane, followed by two extraction cycles with 3% HCl. The organic phase was washed with saturated NaCl, dried over MgSO_4_ and the solvent of the filtrate was evaporated (Yield 97%).

^1^H-NMR (CDCl_3_): δ [ppm]: 1.4–1.5 (4, 9), 1.6–1.7 (8, 10), 1.9 (13), 2.3 (7), 3.2 (1), 3.7 (2), 4.1 (11), 4.6–4.7 (3, 6), 5.6 (12′), 6.1 (12″) 

### 3.4. Synthesis of Poly-(alanine-co-ε-caprolacton)-dimethacrylate (ACM 6:4 and ACM 8:2)

Polymerization of the cyclic peptide for ACM 6:4 was performed as follows. 3-Methyl-morpholine-2,5-dion (7,29 g, 56.5 mmol, 6 eq), ε-Caprolacton (4.2 mL, 37.7 mmol, 4 eq) and Diethyleneglycol (0.89 mL, 9.4 mmol, 1 eq) were added in a flame-dried two-neck round-bottom flask. The educts were dried under vacuum for 2 h and the flask was flushed with argon. Then the flask was heated to 150 °C and Sn(II)EtHex (0.3 mL, 0.9 mmol, 0.1 eq) was added under argon atmosphere, and the flask was sealed under vacuum. The reaction mixture was stirred at 150 °C for 20 h. After that, the flask was cooled down, the product was dissolved in 15 mL dichlormethan and 2 mL methacroylchloride were added (20.7 mmol, 2.2 eq). A dropping funnel was filled with 3.2 mL Triethylamine (22.6 mmol, 2.4 eq) and 15 mL Dichlormethan. The mixture was dropped into the polymer over a time period of 30 min, followed by stirring for 20 h. Then, the product was precipitated five times in cold hexane and dissolved in 200 mL dichlormethane, followed by two extraction cycles with 3% HCl. The organic phase was washed with saturated NaCl, dried over MgSO_4_ and the solvent of the filtrate was evaporated (Yield 95%).

### 3.5. Molecular Characterisation

For the determination of the chemical structures, 1H-NMR experiments were performed on an Agilent spectrometer DD2 500 MHz, VNMRS 400 MHz instrument. For the measurements, deuterated CDCl_3_ or D_2_O were used as solvents and measurement was performed at 27 °C. All experiments were performed under OpenVnmrJ 1.1 and equipped with 5 mm PFG One NMR probe. 

Analysis of temperature-dependent viscosity was performed with a rheometer MCR501 (Anton Paar Germany GmbH, Ostfildern-Scharnhausen, Germany). A plate/plate measurement tool (PP08-SN81066681) with diameter of 1 cm was used. The shear rate was set at 50 s^−1^ and a heating rate of 2 °C min^−1^ in a temperature range between 15 °C and 60 °C was applied.

UV-Vis spectra were measured on a spectrophotometer V-670 (JASCO Deutschland GmbH). UV microcuvettes were used for the measurement with a thickness of 1 cm. For the detection of spectra, the following parameters were used: UV/VIS bandwidth: 1.0 nm, NIR bandwidth: 8.0 nm, Scan speed: 1000 nm/min, Scan mode: Continuous, Data interval: 1.0 nm. 

### 3.6. Contact Angle and Swelling Behavior

The analysis of wettability and swelling properties of the ACM was performed using copolymer discs with a diameter of 7 mm and 1 mm height. For that, 1 g polymer was mixed with 1 wt% IRGACURE^®^369 in Chloroform (100 mg/mL) and the solvent was removed by stirring the mixture at 60 °C overnight. After that, silicon masks with the appropriate dimension were filled with the precursor solution and treated with UV light for 30 min. Thereafter, the discs were placed into an acetone washing solution for 5 d and the solvent was changed two times a day. Before the experiments, the samples were dried overnight at room temperature. 

Determination of water contact angles was carried out with a computer-controlled contact angle measuring system (DCA20, dataphysics instruments GmbH, Filderstadt, Germany) using the sessile drop technique (drops of 3 µL). In order to evaluate the expected hydration effects of the ACM copolymers, both dry and swollen samples were characterized. In order to obtain the wettability of the swollen substrates, polymer discs were incubated in phosphate-buffered saline at 37 °C for 3 d. Prior to the determination of contact angles, excess buffer on the surface was removed. The contact angle data represent an average from 15 measurements at six different samples (n = 90).

For swelling behavior of polymer discs, the diameter (d) of the dry and swollen discs was measured. Swollen samples were prepared as described above. Degree of swelling was calculated as d_swollen_/d_dry_*100%. Measurement was performed for six different samples for each ACM copolymer.

### 3.7. Mechanical Measurement

Samples for mechanical testing were produced by UV curing. Briefly, to 1 g of polymer 1 wt% IRGACURE^®^369 in Chloroform (100 mg/mL) was added. The solvent was removed by stirring the mixture at 60 °C overnight. Silicone masks for samples with a height of 10 mm and a diameter of 6 mm were filled with the polymer and UV cured for 40 min. The longer reaction time in comparison to the preparation of polymer discs is due to the higher sample volume. The UV curing was performed with a Vacuum UV Exposure Unit 2 from proMa systro, which works at a wavelength of 365 nm and a power of 120 W. 

Mechanical analyses were performed in accordance to a measurement and analysis methodology which has been described previously [37]. Stress relaxation data were obtained using TA instruments Electroforce 5200N test instrument. Cylindrical specimens (6 mm diameter, 10 mm height) were compressed up to a decrease in height of 20%. The resulting stress was time resolved over a measuring period of 5 min with a time resolution of 150 ms.

Relaxation data were computed using a viscoelastic model approach consisting of parallel Maxwell elements combined with an elastic Hooke’s element. From a viscoelastic point of view, this model corresponds to an approach where the time constants of the damping elements are given as a sum of two logarithmic normal distributions.

The raw data were fitted 500x independently using an evolutionary algorithm to get 500 individual resulting curves. Based on these data, a so-called master curve was generated containing the median value of the 500 fitting results for each frequency with an uncertainty given by the 50% quantile. 

The master curves were fitted again, on the one hand with a single exponential function and on the other hand with a sum of two exponential functions. This method was chosen to prove whether there is eventually more than one global relaxation region. The obtained fitting results indicate that the polymer system indeed shows two relaxation regions. Following this observation, it is not surprising that the fitting with a sum of two exponential functions shows a residual error several orders of magnitude smaller than fitting the master curves with a single exponential function. 

As characteristic values describing the complex viscoelastic behavior of the investigated materials, it is usual to choose the unrelaxed and the relaxed modulus, the respective half-widths of the relaxation regions and the relative height of the dispersion of the storage modulus. A detailed description of the methodology was published recently [37].

### 3.8. Two-Photon Polymerization (2PP) Setup and Processing

For processing of the material via two-photon polymerization, a novel exposure method was evaluated. In contrast to conventional 2PP approaches, this new method relies on a f-theta scanning objective with a large scan area of 15 mm × 15 mm. This approach was used since the long-term goal is to fabricate scaffolds with a height of 10 mm and a diameter of 7 mm. The fabrication of these large structures is challenging and time consuming, with conventional high NA microscope objectives typically exhibiting a small scan area of less than 1 mm × 1 mm.

The fabrication relies on a bottom-up exposure with a subsequent pulling of the structure out of a reservoir of liquid resist. As a light source, a fs laser with a wavelength of 522 nm, a repetition rate of 63 MHz and a pulse length of 250 fs was used. As ACM 2:8 is in a solid phase at room temperature, its viscosity was reduced during the laser processing by applying a temperature of 50 °C via heating cartridges inserted into the material container. As substrate, a conventional silanized cover glass with dimensions of 18 mm × 18 mm × 0.17 mm was used. After the fabrication, the sample was immersed in acetone for 5 min to remove the unexposed material.

For the analysis of the structures, a scanning electron microscope (Hitachi TM3030, Hitachi High-Tech Europe GmbH, Krefeld, Germany ) and a confocal laser scanning microscope (Olympus LEXT™ OLS4100, OLYMPUS EUROPA SE & CO. KG, Hamburg, Germany) were used.

### 3.9. Cultivation of Mesenchymal Stem Cells

#### 3.9.1. Coating

ACM sample discs were rinsed with double-distilled water. To avoid any hemostatic reaction after blood contact, the ACM polymers were coated with an anti-coagulative heparin layer using the well-established layer-by-layer method. Therefore, the polycation Poly-L-Lysine-Hydrobromide (Sigma Aldrich, Taufkirchen, Germany) and the polyanion Heparin (Sigma Aldrich, Taufkirchen, Germany) were solved in 20 mM Sodium acetate buffer with a concentration of 1 mg/mL each. For the first layer, the samples were dipped in the PLL solution for 5 min, then rinsed two times with ddH_2_O. For the second layer, the samples were dipped in heparin solution for 5 min and then rinsed twice again with ddH_2_O. All dipping and rinsing steps were repeated once. After the last rinsing step, the samples were air dried and sterilized under UV light for 20 min. 

#### 3.9.2. Cell Cultivation

Human mesenchymal stem cells (hMSCs) were cultivated in DMEM (PAN-Biotech GmbH, Aidenbach, Germany, low glucose) with 10% FBS, 0.2% Gentamicin/Amphotericin B and 4 mM Glutamine. Coated and uncoated sample discs were equilibrated in cell culture medium overnight. Then 20,000 cells/cm^2^ were seeded on the samples and incubated for three days at 37 °C and 5% CO_2_.

#### 3.9.3. Staining

Cells on the samples were rinsed two times with PBS and fixed with 4% Paraformaldehyde for 20 min. Cells were stained with Phalloidin488 (Thermo Fisher Scientific Inc., Waltham, Massachusetts, United States ) according to manufacturer information and analyzed with a confocal laser scanning microscope (Carl Zeiss Microscopy Deutschland GmbH, Oberkochen, Germany).

## 4. Conclusions

Scaffold-based approaches in osteochondral tissue engineering rely essentially on the availability of advanced biodegradable polymers without producing adverse degradation products. Within this study, we successfully established a new 2PP processable material platform, based on alanine and ε-caprolacton, with tunable biochemical and biophysical properties. The addition of alanine in the scaffold material is due to increase degradation rate of the polymer and to enhance wettability of the polymer surface, which are drawbacks of pure ε-caprolacton scaffolds. Thus, wettability, which is important for native protein adsorption and initial cell adhesion, can be tailor-made by varying the amount of ε-caprolacton. All synthesized ACM derivatives showed nearly no swelling/shrinkage under physiological conditions, leading to high accuracy of the produced implant. The light penetration increased with increasing wavelength for all polymers, indicating that 2PP structuring at a long wavelength is preferred. The best light penetration was achieved for the mixtures ACM 2:8 and ACM 4:6. For the other two ACM derivatives, light penetration at the 2PP structuring wavelength of 522 nm was between 21% and 25%. Nevertheless, the mechanical properties of ACM 2:8 and ACM 4:6 (400 kPa and 230 kPa for the porous scaffold) match in principle the properties of the surrounding tissue of osteochondral implants and possess, therefore, an excellent suitability for osteochondral reconstruction. First structuring experiments with 2PP showed that it is possible to create stable structures of ACM 2:8. Furthermore, we showed that the material has shown no cytotoxic effect when culturing MSCs on ACM 2:8 polymer discs. In summary, these results demonstrate the high potential of the 2PP-structured ACM polymer system for application in osteochondral tissue engineering. 

## Figures and Tables

**Table 1:** Results from 1H-NMR Analysis of ACM derivates: Calculation of Alanin and caprolacton content as well as molar mass.

Polymer	Alanin (a)	Caprolactone (b)	Molar Mass [g/mol]
ACM 2:8	1.3	7.2	1183
ACM4:6	2.9	5.6	1208
ACM6:4	4.3	3.9	1188
ACM8:2	5.4	2.2	1161

**Table 2:** Comparison of mechanical measurements on material platforms used for scaffolds in bone healing applications.

Material Platform	Application	Methodology	Mechanical Property Range Investigated	Ref.
Poly-Lactide/ Caprolactone Copolymer	bone	compression; 0.5 mmxmin^−1^; 20% strain	180–5500 kPa compressive strength	[38]
PolyHEMA Composite	bone	compression; 5 mmxmin^−1^; 10% strain	60–190 kPa compressive strength	[39]
Cellulose/ Poly-Capro- lactone Composite	bone	tension; 10 mmxmin^−1^; strain not specified	10–18 MPa tensile strength	[40]
Poly-Lactide Composite	bone	microtensile testing; parameter not specified	1.8–6.5 MPa tensile strength	[41]
Poly-Alanine-ε-Caprolacton- Methacrylate Copolymer	bone cartilage	stress relaxation after 20% initial compressive strain	230–400 kPa complex modulus	this study

## Data Availability

The data presented in this study are available on request from the corresponding author.
